# A Novel Adjustment Method for Shearer Traction Speed through Integration of T-S Cloud Inference Network and Improved PSO

**DOI:** 10.1155/2014/865349

**Published:** 2014-11-23

**Authors:** Lei Si, Zhongbin Wang, Xinhua Liu, Yinwei Yang, Lin Zhang

**Affiliations:** ^1^School of Mechatronic Engineering, China University of Mining & Technology, Xuzhou 221116, China; ^2^Xuyi Mine Equipment and Materials R&D Center, China University of Mining & Technology, Huai'an 211700, China

## Abstract

In order to efficiently and accurately adjust the shearer traction speed, a novel approach based on Takagi-Sugeno (T-S) cloud inference network (CIN) and improved particle swarm optimization (IPSO) is proposed. The T-S CIN is built through the combination of cloud model and T-S fuzzy neural network. Moreover, the IPSO algorithm employs parameter automation adjustment strategy and velocity resetting to significantly improve the performance of basic PSO algorithm in global search and fine-tuning of the solutions, and the flowchart of proposed approach is designed. Furthermore, some simulation examples are carried out and comparison results indicate that the proposed method is feasible, efficient, and is outperforming others. Finally, an industrial application example of coal mining face is demonstrated to specify the effect of proposed system.

## 1. Introduction

Currently, the cooperative control of coal mining machines (shearer, scraper conveyers, and hydraulic supports) is becoming a development trend in fully mechanized mining face. As a key factor of cooperative control, the traction speed of shearer has a great influence on the mining efficiency and the working states of other coal mining machines. Therefore, the traction speed should be precisely and reasonably adjusted in a reliable way. However, due to the poor working conditions of coal mining such as narrow space, high coal dust, low visibility, and large noise, shearer operator does not have accurate and timely manner to adjust the traction speed only depending on the vibration noise of shearer mining and manual visualization [1, 2]. This phenomenon cannot ensure shearer work in cooperation with other coal mining machines reasonably and may lead to the problem of low mining efficiency. Moreover, many safe accidents in collieries occurred increasingly frequently. Under this kind of background, the researches on adjustment methods for shearer traction speed have become a challenging and significant research subject.

Due to the randomicity and complexity of underground geological conditions, adjusting traction speed would present the characteristics of complexity, fuzziness, uncertainty, and high risk, and this may affect the coal production or even endanger the operator's life. Therefore, it is necessary to handle the speed accurately and effectively. In the real mining condition, some key index parameters have a strong relationship with shearer traction speed and the relationship is highly nonlinear in nature so that it is hard to develop a comprehensive mathematic model. To deal with this kind of problem, the commonly used methods are fuzzy theory and neural networks [3–5]. Fuzzy neural network (FNN) can combine the advantages of both fuzzy logic in processing vague information and neural network in good learning abilities [6]. It can also handle imprecise information through linguistic expressions. For several decades, FNN has attracted much attention and has been applied in many domains [7].

As a typical type of FNN, Takagi-Sugeno (T-S) type model [8, 9] has been commonly and widely used. However, FNN adopts traditional membership functions (such as trigonometric function, trapezoidal function, and normal function) to describe the subordinate relations [10]. It is difficult to completely reflect the uncertainty only through the precise membership functions. Li et al. proposed a new model, namely, the cloud model on the basis of random mathematics and fuzzy mathematics [11]. Cloud model uses linguistic values to represent the transformation between qualitative concepts and their quantitative representation. Thus, the cloud model can be introduced to replace the membership functions in conventional T-S FNN and then the T-S cloud inference network (T-S CIN) is constituted [12]. However, there are many drawbacks of T-S CIN by common back propagation (BP) algorithm with gradient descent for training, such as being easy to trap into local minimum point and poor ability on global search [13, 14]. In addition, the performance of BP training depends on the initial values of the system parameters, and for different network topologies it has to derive new mathematical expressions for each network layer. Based on the past work on artificial intelligent optimization algorithms, this paper tries to tackle the problem.

Bearing the above observation in mind, we propose an integrated approach based on T-S CIN and improved particle swarm optimization (IPSO) to adjust the shearer traction speed. The remainder of this paper is organized as follows. In Section 2, some related works are outlined based on literatures.  Section 3 describes the integrated approach based on T-S CIN and IPSO algorithm and designs the flowchart of proposed algorithm.  Section 4 provides some simulation examples and carries out the comparison with other methods to verify the feasibility, efficiency, and outperforming of others. An industrial example of mine automation production based on proposed system is demonstrated to specify the application effect in Section 5. Our conclusions are summarized in Section 6.

## 2. Literature Review

Recent publications relevant to this paper are mainly concerned with the streams of learning algorithms for T-S models. In this section, we try to summarize the relevant literatures.

In recent years, many researches have used genetic algorithms (GAs) for the learning of T-S models and attain better performance than BP algorithm [15]. In [16], a hybrid algorithm, combining the advantages of genetic algorithm's strong search capacity and Kalman filter's fast convergence merit, was proposed to construct a “parsimonious” fuzzy model with high generalization ability. Wang et al. proposed a new scheme based on multiobjective hierarchical genetic algorithm extract interpretable rule-based knowledge from data and this method was derived from the use of multiple objective genetic algorithms [17]. In [18], a hybrid system combining a fuzzy inference system and genetic algorithms was proposed to tune the parameters in the Takagi-Sugeno-Kang fuzzy neural network. Lin and Xu proposed a self-adaptive neural fuzzy network with group-based symbiotic evolution method and genetic algorithms were used to adjust the parameters for the desired outputs [19]. In [20], a fuzzy controller design method was proposed based on genetic algorithm to find the membership functions and the rule sets simultaneously. Juang proposed a TSK-type recurrent fuzzy network with a genetic algorithm for control problems [21].

Recently, as a new branch in evolutionary algorithms, particle swarm optimization (PSO) has attracted many researchers' interests [22]. Compared with GA, the PSO has some attractive characteristics, such as simple concept, easy implementation, robustness to control parameters, and computation efficiency when compared with other heuristic optimization techniques. Successful applications of PSO in some optimization problems, such as function optimization and neural network optimization, have demonstrated its potential [23, 24]. The combined method of fuzzy model and PSO algorithm was proposed in [25, 26] and the authors found that PSO algorithm could generate better results for identifying the fuzzy model than GA with the same complex problem. Although PSO algorithm has been developing rapidly, it is relatively inefficient in local search and easy to result in premature convergence. Therefore, some improved approaches and variants of PSO have been reported. Du et al. proposed a novel hybrid learning algorithm based on random cooperative decomposing particle swarm optimization algorithm and discrete binary version of PSO algorithm, and the optimal structure and parameters of T-S FNNs were achieved simultaneously [27, 28]. In [29], a prediction algorithm for traffic flow of T-S fuzzy neural network and improved particle swarm optimization was proposed, and the improved strategy was used to make the algorithm jump out of local convergence by using *t* distribution. Lin proposed a new learning algorithm based on the immune-based symbiotic particle swarm optimization for use in TSK-type neurofuzzy networks to avoid trapping in a local optimal solution and to ensure the search capability of a near global optimal solution [30].

In addition, a cooperative particle swarm optimization (CPSO) algorithm has been proposed based on the notion of coevolution and proven to be more effective than the traditional PSO in most optimization problems [31]. In [32], a powerful cooperative evolutionary particle swarm optimization algorithm based on two swarms with different behaviors to improve the global performance of PSO was proposed. In [33], a novel adaptive cooperative PSO with adaptive search was presented, and the proposed approach combined cooperative learning and PSO to combat curse of dimensionality and control the balance of exploration and exploitation in all the smaller-dimensional subswarms.

According to above analysis, although many improved strategies for PSO have been proposed, they have some common shortcomings summarized as follows. Firstly, most improved IPSO algorithms are hard to get a good tradeoff between global convergence and convergent efficiency. Secondly, it cost long computation time and there is a weak ability in high dimension optimization problems. Finally, there is lack of the effective judge tool to determine whether the particles have gotten into local optimal value or not.

In this paper, an improved PSO algorithm is proposed by employing parameters automation strategy and velocity resetting, and the integrated method based on IPSO learning algorithm and T-S CIN is generated to adjust the shearer traction speed. Some simulation examples and comparison with other methods are carried out, and the proposed approach is proved feasible and efficient.

## 3. The Proposed Method

### 3.1. Cloud Model

The cloud is a model using the linguistic value to represent the uncertainty conversion between a qualitative concept and its quantitative representation. Suppose *U* is a quantitative domain expressed in precise values and *A* is a qualitative concept in *U*. If a quantitative value *x* ∈ *U* is a random realization of the qualitative concept *A* and the membership of *x* to *A*, *μ*(*x*)∈[0, 1], is a random number with a stable tendency: *μ*: *U* → [0,1], ∀*x* ∈ *U*, *x* → *μ*(*x*), then the distribution of *x* on domain *U* is called the cloud and each *x* is called a cloud droplet.

Normal cloud is widely used as a cloud model. We suppose that *R*(*E*_1_, *E*_2_) denotes a one-dimensional normal distribution random function, where *E*_1_ is the expected value and *E*_2_ is the standard deviation. If *x*  (*x* ∈ *U*) and *μ*(*x*) satisfy the equations, which can be expressed as follows:

(1) $$\begin{array}{c} x=R\left(\text{Ex},\text{En}\right),\\ p=R\left(\text{En},\text{He}\right),\\ \mu =\exp\left(-\frac{{\left(x-\text{Ex}\right)}^{2}}{2p^{2}}\right) \end{array}$$

then the distribution of *x* on domain *U* is called the normal cloud. In (1), Ex, En, and He denote the expectation, entropy, and hyper entropy, respectively, which are used to describe the numerical characteristics of cloud. Ex is the expectation of cloud droplets in the distribution of the domain and is the most typical point that represents this qualitative concept. En is the uncertain measurement of the qualitative concept and reflects the relevance of fuzziness and randomness. He is the uncertain measurement of entropy and is determined by the fuzziness and randomness.

**Figure 1:**
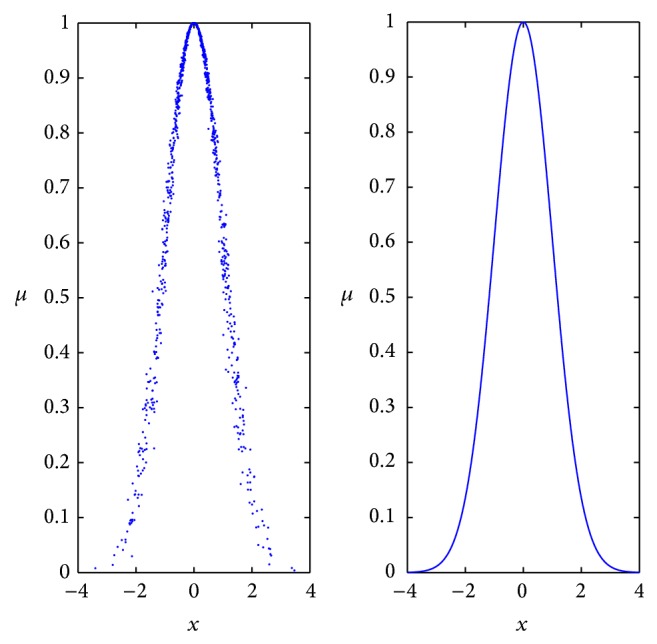
Normal cloud and membership function.

A possible form of normal cloud and membership function, whose linguistic values are close to zero, can be shown as Figure 1. Obviously, membership function is a specific curve. Once the membership function represents the property of fuzziness, it is no longer vague. However, normal cloud is composed of some cloud droplets, which can reflect the fuzziness. The membership is a group of random values with a stable tendency, rather than fixed values. Cloud model is not described through certain functions, therefore, to enhance the processing capacity for uncertainty.

### 3.2. Structure of T-S Cloud Inference Network

**Figure 2:**
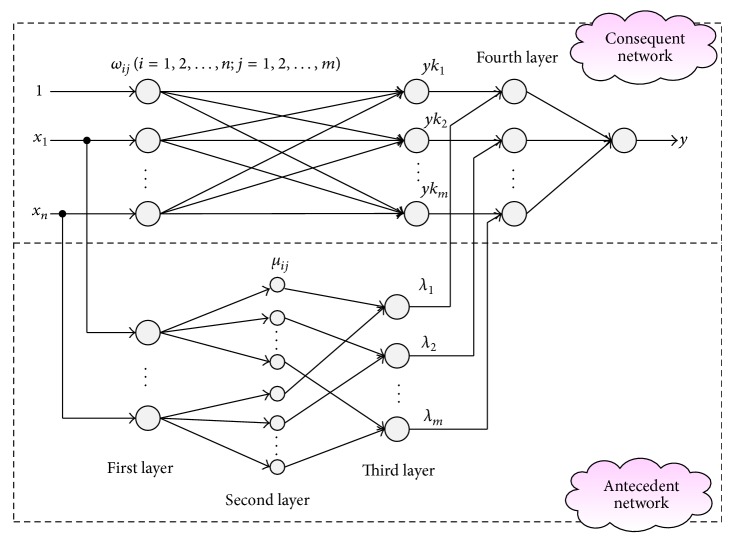
Structure of T-S cloud inference network for the MISO system.

For a multiple-input and single-output (MISO) system, the T-S model can be given as follows: let *X* = [*x*_1_, *x*_2_,…, *x*_*n*_] denote an input vector, where each variable *x*_*i*_ is a fuzzy linguistic variable. The set of linguistic variables for *x*_*i*_ is represented by *T*(*x*_*i*_) = {*A*_*i*_^1^, *A*_*i*_^2^,…, *A*_*i*_^*m*^} (*i* = 1,2,…, *n*), where *A*_*i*_^*j*^ (*j* = 1,2,…, *m*) is the *j*th linguistic value of the input *x*_*i*_. The membership of fuzzy set defined on domain of *x*_*i*_ is *μ*_*ij*_ (*i* = 1,2,…, *n*, *j* = 1, 2,…, *m*). According to [8], the T-S CIN is composed of four layers, which can be divided into two networks: antecedent network and consequent network. The first three layers of this T-S CIN correspond to the antecedent network and the fourth layer is output layer. The structure of T-S CIN can be described as Figure 2.

In Figure 2, the purpose and meaning of each layer can be defined as follows.

*First Layer*. This layer is the input layer of antecedent network and no function is performed in this layer. The nodes are only used to transmit the input values to the second layer. 

*Second Layer*. This layer is the fuzzification layer by the use of cloud model. Nodes in this layer correspond to one linguistic label of the input variables in the first layer. Each node represents a cloud model, which is used to realize the cloud of input variables. In this study, the number of partitions for the cloud is set as *m* and the total number of second layers is *n* × *m*. The degree of membership cloud for input variable *x*_*i*_ can be calculated through the following equations:

(2) μijxi=exp⁡−xi−Exij22pij2,i=1,2,…,n; j=1,2,…,m,

where *p*_*ij*_ = *R*(En_*ij*_, He_*ij*_). 

*Third Layer*. This layer is the cloud inference layer (cloud rule layer). Firing strength of every rule is calculated. Each node describes one cloud rule and is used to match the input vector. The degree that the input vector *X* matches rule Rule_*j*_ can be computed through the following equation:

(3) $$\begin{array}{c} \lambda_{j}=\mu_{1j}\cdot \mu_{2j}\cdots \mu_{nj}=\overset{n}{\underset{i=1}{\prod }}\mu_{ij}\;\left(j=1,2,\ldots ,m\right), \end{array}$$

where *λ*_*j*_ is called the firing strength of rule Rule_*j*_.

*Fourth Layer*. In consequent network, it is a linear relationship between the layers. The hidden layer output of this network can be given through the following equation:

(4) $$\begin{array}{c} yk_{j}=\overset{n}{\underset{i=0}{\sum }}x_{i}\cdot \omega_{ij}\;\left(x_{0}=1;\;j=1,2,\ldots ,m\right), \end{array}$$

where *ω*_*ij*_ is the coefficient of the network.

The output layer sums up all the activated values from the cloud inference rules to generate the overall output *y*, which can be calculated by

(5) $$\begin{array}{c} y=\frac{\sum_{j=1}^{m}\lambda_{j}\cdot yk_{j}}{\sum_{j=1}^{m}\lambda_{j}}. \end{array}$$

### 3.3. Learning Algorithm for T-S CIN

According to the principle of T-S CIN, the structure and parameters manly include the expectation Ex_*ij*_, entropy En_*ij*_, hyper entropy He_*ij*_ of cloud model, and coefficient *ω*_*ij*_ of consequent network. Conventional learning algorithm for T-S CIN is the gradient descent method. However, the initial values of gradient descent method have a great influence on the learning effect of network and this method is easy to fall into local minimum. In this paper an improved particle swarm optimization algorithm (IPSO) is proposed as the learning algorithm to optimize the structure and parameters of T-S CIN.

**Figure 3:**
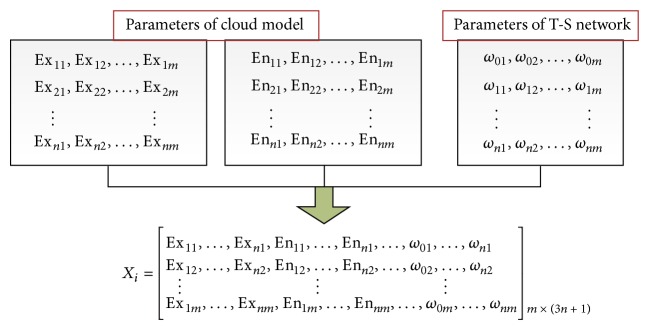
Encoding of a particle location.

The basic particle swarm optimization algorithm (PSO) is that a swarm of particles are initialized randomly in the solution space and each particle motions in a certain rule to explore the optimal solution after several iterations. It has two attributes of position and velocity. The position of the *i*th particle is *X*_*i*_ and the velocity can be denoted by *V*_*i*_. In T-S CIN, the parameter of hyper entropy He_*ij*_ is the uncertain measurement of entropy and depends on the actual situation. In this paper, He_*ij*_ is set as He_*ij*_ = En_*ij*_/10. Thus, other parameters should be optimized through PSO. The location of a particle *X*_*i*_ corresponding to T-S CIN can be encoded as Figure 3.

Therefore, the position and the velocity of the *i*th particle can be given as

(6) $$\begin{array}{c} X^{i}=\left[\begin{array}{c} x_{11}^{i},x_{12}^{i},\ldots ,x_{1,3n+1}^{i}\\ x_{21}^{i},x_{22}^{i},\ldots ,x_{2,3n+1}^{i}\\ \vdots \\ x_{m1}^{i},x_{m2}^{i},\ldots ,x_{m,3n+1}^{i} \end{array}\right],\\ V^{i}=\left[\begin{array}{c} v_{11}^{i},v_{12}^{i},\ldots ,v_{1,3n+1}^{i}\\ v_{21}^{i},v_{22}^{i},\ldots ,v_{2,3n+1}^{i}\\ \vdots \\ v_{m1}^{i},v_{m2}^{i},\ldots ,v_{m,3n+1}^{i} \end{array}\right]. \end{array}$$

Particles are updated through tracking two “extremums” in each iteration. One is the individual optimal solution *P* ^*i*^ = [*p*_*jl*_^*i*^]_*m*×(3*n* + 1)_ found by the particle itself and another is the global optimal solution *P* ^*g*^ = [*p*_*jl*_^*g*^]_*m*×(3*n* + 1)_ found by the particle population. The specific iteration formulas can be expressed as follows:

(7) vjlik+1=wkvjlik+c1r1pjlik−xjlik+c2r2pjlgk−xjlik,xjlik+1=xjlik+vjlik+1,

where *k* is the current iteration times; *i* = 1, 2,…, *M*,  *M* is the number of particles; *j* = 1, 2, …, *m* and *l* = 1, 2, …, 3*n* + 1; *c*_1_ and *c*_2_ are the acceleration coefficients; *r*_1_ and *r*_2_ are uniformly distributed random numbers in the range (0,1). The velocity vector *V* is limited to the range [−*V*_max⁡_, *V*_max⁡_] to reduce the likelihood of the particle leaving the search space and the position vector *X* is clamped to the range [*X*_min⁡_, *X*_max⁡_], which can be determined according to practical problem and *V*_max⁡_ is usually chosen to be *α* × *X*_max⁡_, with *α* ∈ [0.1, 1.0]; *ω*_*k*_ is the current inertia weight.

Shi and Eberhart [34] proposed a linearly varying inertia weight (*w*_*k*_) over the course of generations, which significantly improves the performance of PSO and can be updated by the following equation:

(8) $$\begin{array}{c} w_{k}=\left(w_{\max}-w_{\min}\right)\frac{T-k}{T}+w_{\min}, \end{array}$$

where *w*_max⁡_ and *w*_min⁡_ are the maximum and minimum of inertia weight; *T* is the maximum number of allowable iterations. The empirical studies in [34] indicated that the optimal solution can be improved by varying the value of *w*_*k*_ from 0.9 at the beginning of the evolutionary process to 0.4 at the end of the evolutionary process for most problems.

Although the version of PSO based on the time-varying inertia weight is capable of locating a good solution with a significantly faster velocity, the ability to fine-tune the optimum solution is comparatively weak, mainly due to the lack of diversity at the end of the evolutionary process. Observed from (7), the particles tend to the optimal solution through two stochastic components: one is the cognitive component and another is the social component. Thus, proper control of the two components is urgently needed and effective for searching for the optimum solution. In this paper, a version of PSO based on time-varying acceleration coefficients is presented to adjust the components by decreasing *c*_1_ and increasing *c*_2_ with time. Based on empirical studies, Ratnaweera et al. [35] have observed that the optimal solutions on most of the benchmarks can be improved by decreasing *c*_1_ from 2.5 to 0.5 and increasing *c*_2_ from 0.5 to 2.5 over the full range of the search. Therefore, the varying scheme of *c*_1_ and *c*_2_ can be given as follows:

(9) $$\begin{array}{c} c_{1}=2.5-\left(2.5-0.5\right)\cdot \frac{k}{T},\\ c_{2}=\left(2.5-0.5\right)\cdot \frac{k}{T}+0.5. \end{array}$$

At the beginning of the search, a large cognitive component and a small social component are assigned to guarantee the particles' moving around the search space. On the other hand, a small cognitive component and a large social component allow the particles to converge to the global optimum in the latter of the search.

PSO can quickly find a good local solution but it sometimes suffers from stagnation without an improvement and then traps in the local optimal solution. In this study, the fitness variance is adopted to measure whether PSO gets into local optimum, which can be calculated as follows:

(10) $$\begin{array}{c} \sigma^{2}=\frac{1}{M}\overset{M}{\underset{i=1}{\sum }}{\left[\frac{1}{f_{\Delta }}\left(f_{i}-\frac{1}{M}\overset{M}{\underset{i=1}{\sum }}f_{i}\right)\right]}^{2}, \end{array}$$

where *f*_*i*_ denotes the fitness of the *i*th particle; *f*_Δ_ denotes the normalized factor. The fitness function and *f*_Δ_ can be calculated as follows:

(11) $$\begin{array}{c} f_{i}=\frac{1}{q}\overset{q}{\underset{s=1}{\sum }}{\left(y_{s}-Y_{s}\right)}^{2},\\ f_{\Delta }=\max\left\{1,\max\left\{\left|f_{i}-\frac{1}{M}\overset{M}{\underset{i=1}{\sum }}f_{i}\right|\right\}\right\}, \end{array}$$

where *q* is the total number of training samples; *y*_*s*_ is the network output of the *s*th training sample; *Y*_*s*_ is the expected output. Thus, *f*_*i*_ is the normalized mean squared error (MSE) of the individual *i* on the training set.

The fitness variance *σ* ^2^ is the symbol of particles convergence degree. When *σ* ^2^ is smaller than a specified value *σ*_min⁡_^2^, the algorithm is considered as falling into precocity. Therefore, to avoid this drawback of basic PSO, a mutation mechanism based on resetting the velocity is proposed to enable particles to have a new momentum. Under this new strategy, when *σ* ^2^ < *σ*_min⁡_^2^, each particle *i* will be selected by a predefined probability from the population, and then a random perturbation is added to each dimension *v*_*jl*_^*i*^ (selected by a predefined probability) of velocity vector *V* ^*i*^ of the selected particle *i*. The pseudocode of resetting velocity can be given as in Pseudocode 1, where *p*_1, *p*_2, and *p*_3 are separately generated and uniformly distributed random numbers in range (0, 1).

### 3.4. Flowchart of Proposed Method

**Figure 4:**
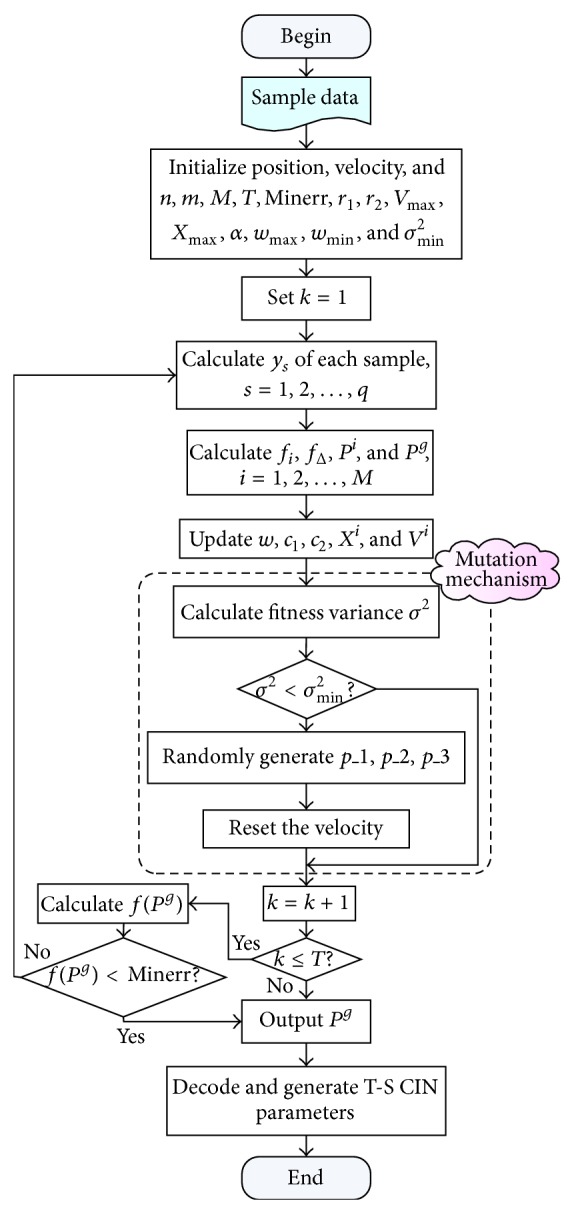
Flowchart of proposed method.

With above specific treatment, structure and parameters of the T-S CIN evolution can be implemented by IPSO. According to above description about the learning algorithm for T-S CIN, the proposed approach is an iterative algorithm and can be coded easily on the computer, and the flowchart can be summarized as shown in Figure 4.

## 4. Simulation Examples

In this section, an example on the adjustment of shearer traction speed is provided to validate the proposed method. The aim of this study is to improve the accuracy and efficiency of identification for traction speed. Furthermore, the example can be divided into three main stages. Firstly, according to the working principle of shearer, the level of traction speed and mainly evaluation indexes can be determined and the sample can be established reasonably. Secondly, according to the obtained evaluation indexes, the T-S CIN model can be constructed. Thirdly, the constructed standard T-S CIN, the T-S CIN with PSO, IPSO, and traditional T-S FNN, the T-S FNN with PSO, IPSO optimization are, respectively, tested with the same training and test samples to compare the accuracy and efficiency in adjustment of shearer traction speed.

### 4.1. Sample Preparation

**Figure 5:**
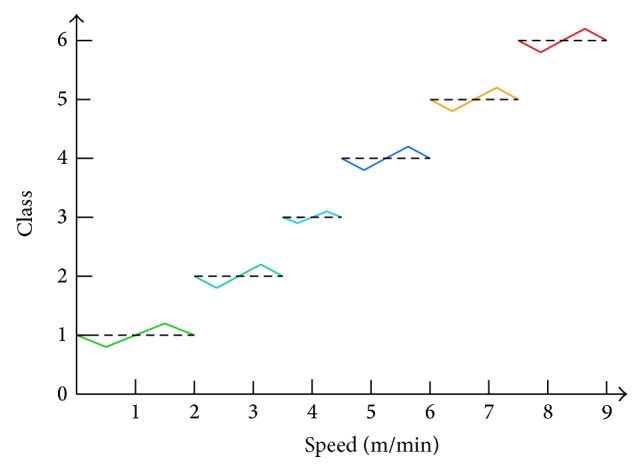
Redefined levels of traction speed.

In a fully mechanized coal mining face, the adjustment of shearer traction speed should consider the coordination with other coal mining equipment (scraper conveyor and hydraulic support). After the analysis of shearer working principle, the evaluation indexes of traction speed mainly consist of cutting motor current (CMC), cutting motor temperature (CMT), traction motor current (TMC), traction motor temperature (TMT), scraper conveyor current (SCC), and scraper conveyor speed (SCS). For a fixed shearer of MG 300/730-WD, the adjusting range of traction speed is 0~9.0 m/min. The levels of traction speed are reasonably partitioned and can be applied in controlling shearer. Based on the empirical studies, the speed levels can be divided as 0~2.0 m/min (Class I), 2.0~3.5 m/min (Class II), 3.5~4.5 m/min (Class III), 4.5~6.0 m/min (Class IV), 6.0~7.5 m/min (Class V), and 7.5~9.0 m/min (Class VI). However, as the information in the database is collected after the workers operate the coal mining equipment, the information maybe not very ideal and practical. Therefore, a threshold of 0.2 is introduced to express the subjective factors, and the traction speed levels from the database can be processed and described as Figure 5.

Taken Class 1 (Class I) as an example, the level of speed 0~2 m/min can be redefined as follows:

(12) $$\begin{array}{c} \text{Class}{\left(\text{Sp}\right)}_{1}^{\text{New}}=\left\{\begin{array}{cc} -0.4\text{Sp}+1, & 0<\text{Sp}\leq 0.5,\\ 0.4\text{Sp}+0.6, & 0.5<\text{Sp}\leq 1.5,\\ -0.4\text{Sp}+1.8, & 1.5<\text{Sp}\leq 2, \end{array}\right. \end{array}$$

where Sp is the current traction speed of shearer. In the same way, the redefined functions for other speeds can also be obtained easily.

**Figure 6:**
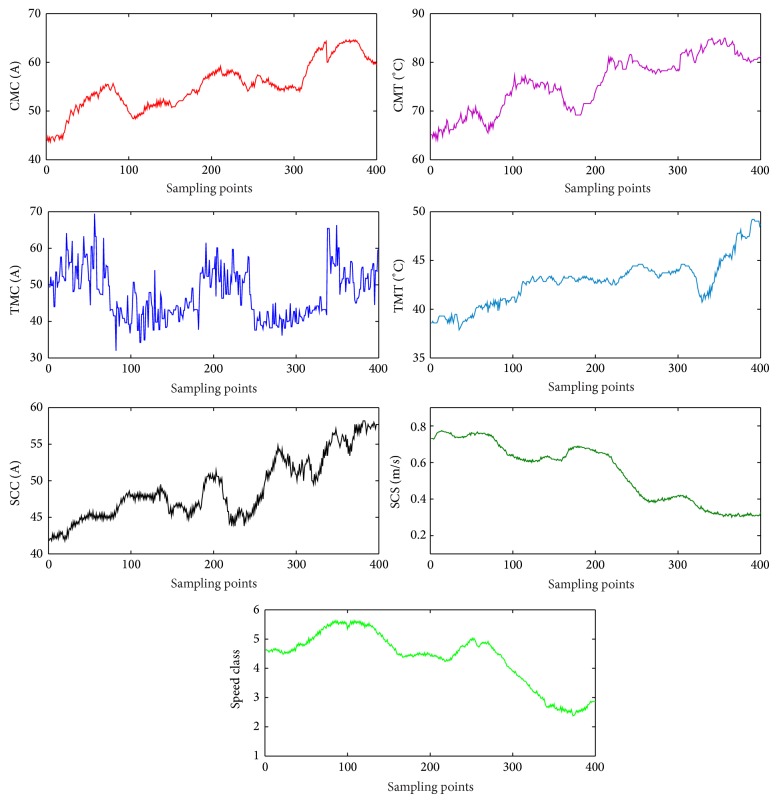
Sample data of this example.

According to the information database acquired from the 2215 coal face in Changcun Coal Mine of Yima Coal Industry Group Co., 400 groups of samples are randomly extracted and rearranged as shown in Figure 6.

### 4.2. Parameters Selection for Proposed Method

There are some parameters in IPSO which need to be specified by the user. However, it is unnecessary to tune all these parameters for the sample data because IPSO is not very sensitive to them. Therefore, these parameters are set as the number of particles *M*  (50); the maximum number of allowable iterations *T*  (500); the position and velocity range of particles ([−1, 1]); the initial acceleration coefficients *c*_1_ and *c*_2_ of IPSO (2.5 and 0.5); the inertia weights *w*_max⁡_ and *w*_min⁡_ of IPSO (0.9 and 0.4); the termination error Minerr  (0.0001); the minimum fitness variance for mutation *σ*_min⁡_^2^  (0.001).

The structure of T-S CIN is determined by the sample data. In this simulation example, the input data of T-S CIN is 6-dimensional and output data is 1-dimensional. Thus, *n* = 6 and *m* can be set as 12. Other parameters including expectation Ex_*ij*_, entropy En_*ij*_, hyper entropy He_*ij*_, and coefficient *ω*_*ij*_ can be optimized through IPSO.

### 4.3. Simulation Results

The sample data in Figure 6 should be normalized firstly and are randomly split into a training data set containing 350 samples and a testing data set containing the remaining 50 samples, which is only used to verify the accuracy and the effectiveness of the trained T-S CIN model.

**Figure 7:**
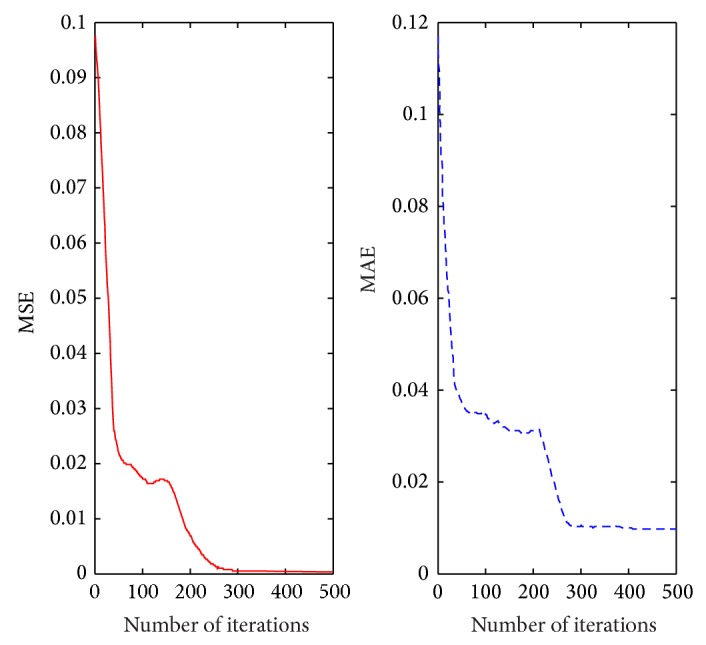
The learning curves of T-S CIN model based on IPSO.

The relevant parameters are given as Section 4.3 described. The proposed method runs 10 times and the mean values are regarded as the final results. The performance criterion of T-S CIN can be measured by the mean squared absolute error (MSE) and the mean absolute error (MAE) between the predicted outcome and the actual outcome. The learning curves with MSE and MAE of T-S CIN model based on IPSO can be shown in Figure 7.

As shown in Figure 7, after the IPSO-based T-S CIN model is trained for 500 times, MSE of the training samples can reach 0.00065 and MAE can reach 0.00987. Actually, the values of MSE and MAE basically keep stable at the times of 280, which can show good convergence performance of proposed method.

**Figure 8:**
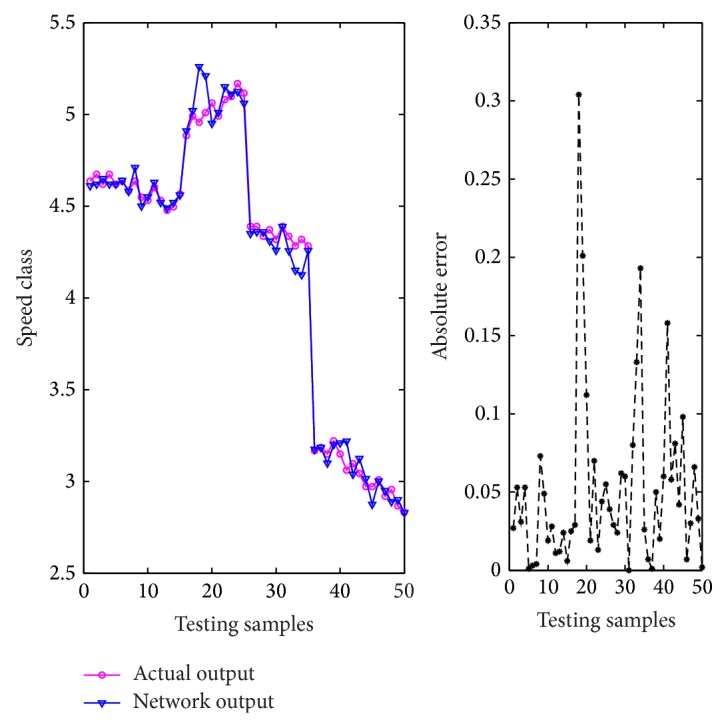
Comparison of network output and actual output.

After the training phase, a T-S CIN model can be obtained. In order to verify the accuracy of the model, the remaining 50 samples are utilized to test its performance. The prediction errors and deviation comparison diagrams of the network output and actual output are given as Figure 8. As shown in Figure 8, the MSE and MAE of testing samples are 0.006118 and 0.0346, respectively, showing good generalization performance. Furthermore, the mean relative error and maximum relative error are 1.23% and 5.78%, which satisfies the accuracy requirement.

### 4.4. Comparison with Other Methods

**Figure 9:**
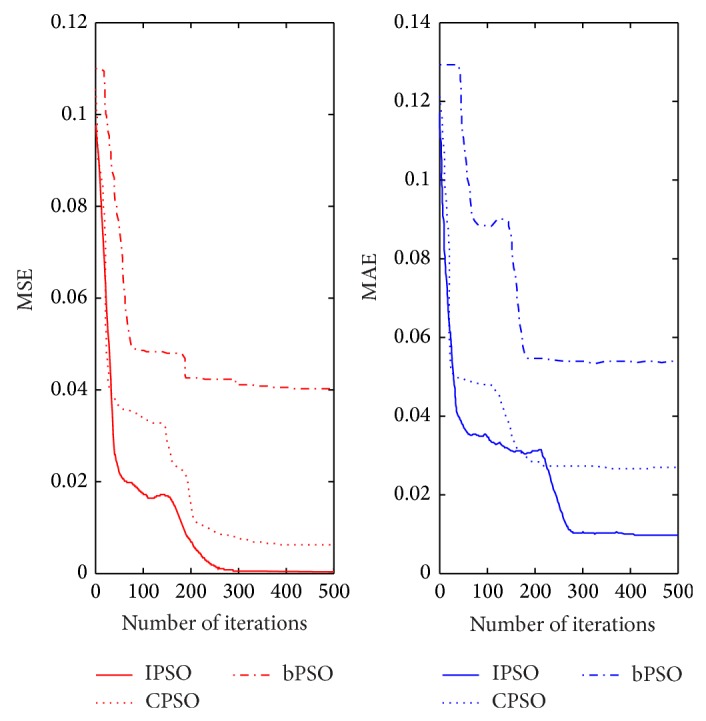
The compared learning curves with MSE and MAE of T-S CIN based on bPSO, CPSO, and IPSO.

In order to indicate the meliority of T-S CIN integrating IPSO, the T-S CINs based on the basic PSO (bPSO), CPSO, and IPSO are provided to solve the problem of above example. The training samples and testing samples are the same. The configurations of simulation environment for three algorithms are uniform and the relevant parameters are in common with above example. The compared learning curves with MSE and MAE of T-S CIN models based on bPSO, CPSO, and IPSO can be shown in Figure 9 and some performance criterions are listed in Table 1, where 50_MSE and 50_MAE are the values of MSE and MAE in the stage of 50 iterations. Furthermore, MRE and MaxRE denote the mean relative error and maximum relative error of the network output and actual output.

Seen from Figure 9 and Table 1, the declining velocity of the error of CPSO and IPSO is faster than that of bPSO during the training phase. The MAE of IPSO-based T-S CIN gets to <0.05 for 30 iterations and the MSE of training phase reaches a stable phase for 300 iterations. However, the training errors of MAE with the bPSO, CPSO-based T-S CIN model are still 0.05026 and 0.1293 for 30 iterations. In the testing phase, the test sample error of bPSO, CPSO-based T-S CIN is much larger than the same input conditions of proposed method. By analysis, the criterions of CPSO-based T-S CIN are more excellent than these of other methods both in the training stage and in the testing stage, which proves the effectiveness and feasibility of proposed method.

**Figure 10:**
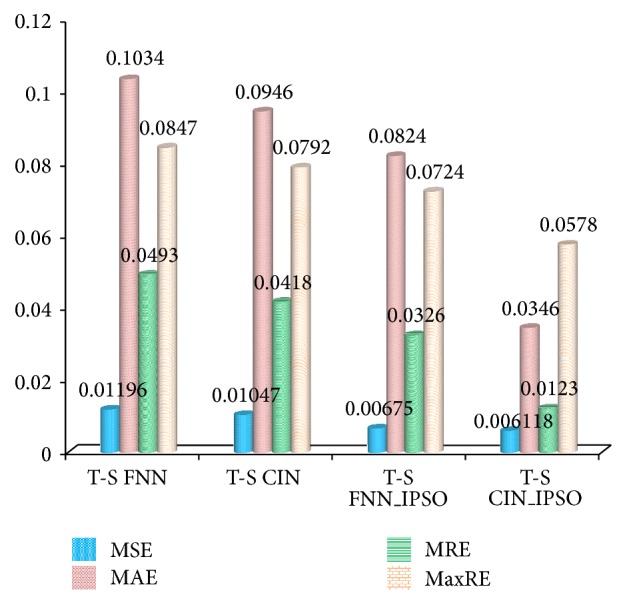
Comparison of the testing results based on four algorithms.

In order to verify the superiority of T-S CIN (T-S NN coupling cloud model), the sample data in Figure 6 are used to test the performance of T-S CIN and conventional T-S FNN, and the proposed IPSO is also integrated with the two networks. Thus, four algorithms are developed, marked as T-S FNN, T-S CIN, T-S FNN_IPSO, and T-S CIN_IPSO. The configurations of simulation environment for four algorithms are uniform and the parameters are in common with above simulation example. The training samples and testing samples of these algorithms should keep consistent. In order to avoid the random error, each algorithm runs 10 times and calculated the average values. The comparison diagram of different testing results is shown in Figure 10.

As Figure 10 illustrated, the prediction errors of T-S CIN are obviously smaller than these of T-S CIN. Through the application of cloud model replacing the membership function in T-S model, the processing capacity for the uncertainty of the problem can be enhanced and the T-SCIN performs with lower MSE, MAE, MRE, and MaxRE. Furthermore, the compared results of coupling IPSO algorithm verify the outperforming others of proposed method.

### 4.5. Further Discussion

In order to further compare and analyze the overall performance of T-S CIN based on IPSO, CPSO, and PSO optimization with the optimal solution (the actual value), the same 400 samples are experimented. In this example, a certain number of samples, denoted by training-size (*T*_size_), are randomly selected from the data as the training samples and 50 samples are randomly selected from the remaining 400 − *T*_size_ samples as the testing samples. Each neural network is then trained and tested 50 times and the average result is recorded as the final result. In this study, the training-size of the example varies over *T*_size_ = 50, 80, 110,…, 350. That is to say, we run several trials over the networks with training-size ranging from 50 to 350. According to [36], the relative error |*y* − *Y* | /*Y* (where *y* is the network output and *Y* is the expected output) is chosen as the metric to express the result as a proportion of the optimal solution (the actual value).

**Figure 11:**
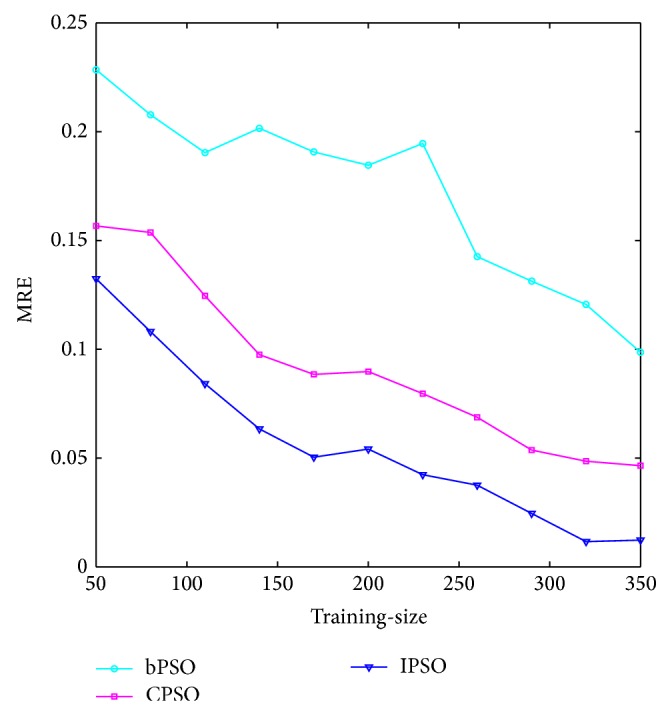
The changes of MRE with different training-sizes.

Figure 11 plots the means of this metric (MRE) for each trial as a function of problem size *T*_size_. It can be seen that for all trials the MRE decreases nonlinearly with *T*_size_ and the T-S CIN based on IPSO optimization outperforms T-S CIN based on CPSO optimization, which in turn outperforms T-S CIN based on bPSO optimization for all *T*_size_.

From Figure 11, it is obvious that the deviation of T-S CIN based on IPSO optimization is the smallest across different training-sizes, which means that the T-S CIN based on IPSO optimization is more stable and robust, and owns stronger generalization ability than T-S CIN based on CPSO and PSO optimization regardless of the training-size. Therefore, the T-S CIN based on IPSO optimization can obtain a relative high accuracy to provide an effective support tool for fuzzy and uncertain adjustment for shearer traction speed.

## 5. Industrial Application

**Figure 12:**
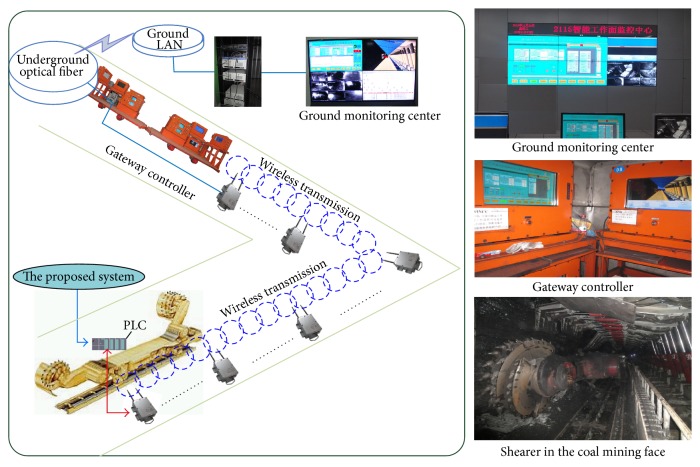
Industrial application example of proposed method.

In this section, a system based on proposed approach has been developed and applied in the field of coal mining face as shown in Figure 12.

As Figure 12 has shown, the “Gateway controller” and “Ground monitoring center” are used to control and monitor the shearer working parameters, which are located underground and on the ground, respectively. The proposed system is uploaded into the PLC (programmable logic controller) installed on the shearer and the speed level can be obtained. The traction speed of shearer can be adjusted through the speed level with Figure 5. The parameters of shearer are transferred into the “Gateway controller” through the wireless network. The “Ground monitoring center” receives these data through the communication of the underground optical fiber and the ground LAN.

**Figure 13:**
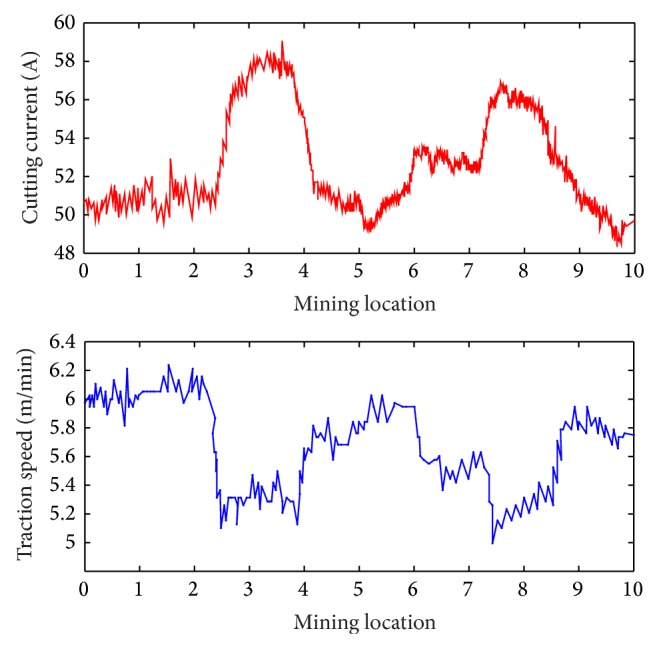
Application effect of proposed system.

For the shearer, the aim of adjusting traction speed is to ensure shearer mine coal smoothly and efficiently when shearer cuts the coal with gangue. In order to illustrate the application effect of proposed system, the shearer operator records the location of cutting the coal or the coal with gangue. This effect can be perfectly reflected through the changes of cutting motor current. In this experiment, the cutting motor current is collected every 1 Hz and the collected data are transmitted to the “Gateway controller” and “Ground monitoring center.” The change curve of cutting motor current is plotted to illustrate the application effect of proposed system, as shown Figure 13.

Seen from   Figure 13, the cutting currents at the location of 2.5 m to 4.0 m and 7.3 m to 8.2 m are a little higher than other locations because shearer cut the coal with gangue, and the corresponding traction speeds are adjusted timely to lower levels through the proposed system. The application effect indicates that the system based on proposed method can provide a feasible strategy for safe and efficient coal mining.

## 6. Conclusions

In this paper, a novel adjustment method for shearer traction speed is proposed, which is based on T-S CIN with integrating IPSO algorithm. IPSO enables T-S CIN to dynamically evolve its parameters by using a specific individual representation and evolutionary scheme. To improve efficiency of PSO in global search and fine-tuning of the solutions, parameter automation adjustment strategy and velocity resetting are used in IPSO algorithm. To demonstrate the performance of proposed method, some simulation examples are provided and some comparisons with other methods are carried out. The results verify that the IPSO-based T-S CIN is an effective support tool for fuzzy and uncertain traction speed adjusting of shearer.

## Figures and Tables

**Pseudocode 1:**
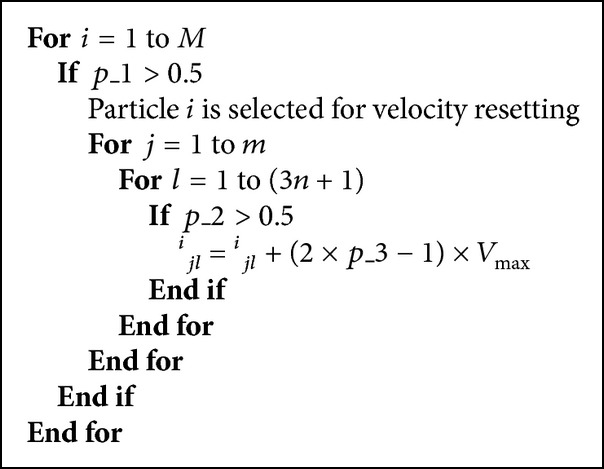


**Table 1:** The compared criterions of T-S CIN based on bPSO, CPSO, and IPSO.

Method	Training phase				Testing phase			
	MSE	MAE	50_MSE	50_MAE	MSE	MAE	MRE	MaxRE
bPSO	0.04024	0.05396	0.07361	0.11573	0.09726	0.2324	9.86%	17.36%
CPSO	0.00626	0.02698	0.03657	0.04956	0.01853	0.0954	4.65%	9.57%
IPSO	0.00065	0.00987	0.02196	0.03779	0.006118	0.0346	1.23%	5.78%

## References

[1] Si L., Wang Z. B., Liu X. H., Tan C. (2014). Cutting path planning of shearer based on prediction of coal seam distribution. *Journal of China University of Mining & Technology*.

[2] Wang Z., Si L., Tan C., Liu X. (2014). A novel approach for shearer cutting load identification through integration of improved particle swarm optimization and wavelet neural network. *Advances in Mechanical Engineering*.

[3] Otadi M. (2012). System of fully fuzzy nonlinear equations with fuzzy neural network. *Neural Computing and Applications*.

[4] Mosavi M. R. (2006). Comparing DGPS corrections prediction using neural network, fuzzy neural network, and Kalman filter. *GPS Solutions*.

[5] Wang Z. X., Sun Y. G., Zhang Q., Qin J., Sun X. W., Shen H. Y. (2006). Research on fuzzy neural network algorithms for nonlinear network traffic predicting. *Optoelectronics Letters*.

[6] Chen Y., Yang B., Abraham A., Peng L. (2007). Automatic design of hierarchical Takagi-Sugeno type fuzzy systems using evolutionary algorithms. *IEEE Transactions on Fuzzy Systems*.

[7] Yoon Y., Guimaraes T., Swales G. (1994). Integrating artificial neural networks with rule-based expert systems. *Decision Support Systems*.

[8] Takagi T., Sugeno M. (1985). Fuzzy identification of systems and its applications to modeling and control. *IEEE Transactions on Systems, Man and Cybernetics*.

[9] Lin F.-J., Lin C.-H., Shen P.-H. (2001). Self-constructing fuzzy neural network speed controller for permanent-magnet synchronous motor drive. *IEEE Transactions on Fuzzy Systems*.

[10] Jang J.-S. R. (1993). ANFIS: adaptive-network-based fuzzy inference system. *IEEE Transactions on Systems, Man and Cybernetics*.

[11] Li D. Y., Liu C. Y., Liu L. Y. (2004). Study on the universality of the normal cloud mode. *Engineering Sciences*.

[12] Gan S. Q., Liu H. P., Shen Z. J. (2005). An improved T-S fuzzy neural network. *Control Engineering of China*.

[13] Pedrycz W., Reformat M. (2003). Evolutionary fuzzy modeling. *IEEE Transactions on Fuzzy Systems*.

[14] Zhang X., Zhao W., Zhang S., Xu T. (2013). Method of flatness pattern recognition based on improved T-S cloud inference network. *Journal of Central South University (Science and Technology)*.

[15] Oh S.-K., Pedrycz W., Park H.-S. (2003). Hybrid identification in fuzzy-neural networks. *Fuzzy Sets and Systems*.

[16] Wang L., Yen J. (1999). Extracting fuzzy rules for system modeling using a hybrid of genetic algorithms and Kalman filter. *Fuzzy Sets and Systems*.

[17] Wang H., Kwong S., Jin Y., Wei W., Man K. F. (2005). Multi-objective hierarchical genetic algorithm for interpretable fuzzy rule-based knowledge extraction. *Fuzzy Sets and Systems*.

[18] Tang A. M., Quek C., Ng G. S. (2005). GA-TSKfnn: parameters tuning of fuzzy neural network using genetic algorithms. *Expert Systems with Applications*.

[19] Lin C.-J., Xu Y.-J. (2006). A self-adaptive neural fuzzy network with group-based symbiotic evolution and its prediction applications. *Fuzzy Sets and Systems*.

[20] Homaifa A., Mccormick E. (1995). Simultaneous design of membership functions and rule sets for fuzzy controllers using genetic algorithms. *IEEE Transactions on Fuzzy Systems*.

[21] Juang C.-F. (2002). A TSK-type recurrent fuzzy network for dynamic systems processing by neural network and genetic algorithms. *IEEE Transactions on Fuzzy Systems*.

[22] Kennedy J., Eberhart R. Particle swarm optimization.

[23] Damodaran P., Diyadawagamage D. A., Ghrayeb O., Vélez-Gallego M. C. (2012). A particle swarm optimization algorithm for minimizing makespan of nonidentical parallel batch processing machines. *International Journal of Advanced Manufacturing Technology*.

[24] Deng A. D., Zhao L., Wei W. (2009). The application of wavelet neural network optimized by particle swarm in localization of acoustic emission source. *Neural Information Processing*.

[25] Khosla A., Kumar S., Aggarwal K. K. A framework for identification of fuzzy models through particle swarm optimization.

[26] Khosla A., Kumar S., Ghosh K. R. A comparison of computational efforts between particle swarm optimization and genetic algorithm for identification of fuzzy models.

[27] Du G., Jiang Z., Diao X., Ye Y., Yao Y. (2012). Variances handling method of clinical pathways based on t-s fuzzy neural networks with novel hybrid learning algorithm. *Journal of Medical Systems*.

[28] Du G., Jiang Z., Diao X., Yao Y. (2012). Knowledge extraction algorithm for variances handling of CP using integrated hybrid genetic double multi-group cooperative PSO and DPSO. *Journal of Medical Systems*.

[29] Hou Y., Zhao H. (2014). Traffic flow prediction based on T-S fuzzy neural network optimized improved particle swarm optimization. *Computer Engineering and Applications*.

[30] Lin C. J. (2008). An efficient immune-based symbiotic particle swarm optimization learning algorithm for TSK-type neuro-fuzzy networks design. *Fuzzy Sets and Systems*.

[31] van den Bergh F., Engelbrecht A. P. (2004). A cooperative approach to particle swarm optimization. *IEEE Transactions on Evolutionary Computation*.

[32] Chen D., Zhao C., Zhang H. (2011). An improved cooperative particle swarm optimization and its application. *Neural Computing and Applications*.

[33] Wang L. (2013). An improved cooperative particle swarm optimizer. *Telecommunication Systems*.

[34] Shi Y., Eberhart R. C. Empirical study of particle swarm optimization.

[35] Ratnaweera A., Halgamuge S. K., Watson H. C. (2004). Self-organizing hierarchical particle swarm optimizer with time-varying acceleration coefficients. *IEEE Transactions on Evolutionary Computation*.

[36] Coffin M., Saltzman M. J. (2000). Statistical analysis of computational tests of algorithms and heuristics. *INFORMS Journal on Computing*.

